# Virale Zoonosen in Deutschland aus der One Health-Perspektive

**DOI:** 10.1007/s00103-023-03709-0

**Published:** 2023-06-01

**Authors:** Rainer G. Ulrich, Stephan Drewes, Viola Haring, Jessica Panajotov, Martin Pfeffer, Dennis Rubbenstroth, Johannes Dreesman, Martin Beer, Gerhard Dobler, Sascha Knauf, Reimar Johne, Merle M. Böhmer

**Affiliations:** 1 Institut für neue und neuartige Tierseuchenerreger, Friedrich-Loeffler-Institut, Bundesforschungsinstitut für Tiergesundheit, Südufer 10, 17493 Greifswald-Insel Riems, Deutschland; 2 Fachgruppe Viren in Lebensmitteln, Bundesinstitut für Risikobewertung, Berlin, Deutschland; 3 Institut für Tierhygiene und Öffentliches Veterinärwesen, Universität Leipzig, Leipzig, Deutschland; 4 Institut für Virusdiagnostik, Friedrich-Loeffler-Institut, Bundesforschungsinstitut für Tiergesundheit, Greifswald-Insel Riems, Deutschland; 5 Niedersächsisches Landesgesundheitsamt, Hannover, Deutschland; 6 Abteilung Virologie und Rickettsiologie, Institut für Mikrobiologie der Bundeswehr, München, Deutschland; 7 Institut für Internationale Tiergesundheit/One Health, Friedrich-Loeffler-Institut, Bundesforschungsinstitut für Tiergesundheit, Greifswald-Insel Riems, Deutschland; 8 Landesinstitut Gesundheit II – Task Force Infektiologie, Bayerisches Landesamt für Gesundheit und Lebensmittelsicherheit (LGL), München, Deutschland; 9 Institut für Sozialmedizin und Gesundheitssystemforschung, Otto-von-Guericke Universität, Magdeburg, Deutschland

**Keywords:** Hantaviren, Hepeviren, Bornaviren, Flaviviren, Influenzaviren, Hantaviruses, Hepeviruses, Bornaviruses, Flaviviruses, Influenza viruses

## Abstract

COVID-19-Pandemie und gehäuftes Auftreten von Mpox-Erkrankungen (Affenpocken) außerhalb Afrikas haben die Verletzlichkeit der Bevölkerung für aus dem Tierreich stammende Krankheitserreger deutlich werden lassen. Darüber hinaus haben in den vergangenen Jahren weitere virale Zoonoseerreger an Bedeutung gewonnen.

Der vorliegende Übersichtsartikel beleuchtet anhand von 6 meldepflichtigen viralen Zoonoseerregern beispielhaft die Notwendigkeit der One Health-Herangehensweise, um die Epidemiologie der Erkrankungen verstehen zu können und Handlungsempfehlungen für den öffentlichen Gesundheitsdienst abzuleiten. Dabei wird die Bedeutung von Umweltfaktoren, Reservoiren und Vektoren betont, die Erkrankungen bei Nutz- und Wildtieren werden analysiert sowie das Auftreten und die Häufigkeit von Erkrankungen bei der Bevölkerung beschrieben. Die hier ausgewählten Erreger unterscheiden sich in den Reservoiren und der Rolle von Vektoren für die Übertragung, den Auswirkungen der Infektionen auf landwirtschaftliche Nutztiere und den beim Menschen beobachteten Krankheitsbildern. Neben bereits lange in Deutschland bekannten Zoonoseerregern werden auch Erreger betrachtet, die erst kürzlich eingetragen wurden bzw. deren Zoonosepotenzial vor Kurzem erstmals gezeigt worden ist.

Bei den hier behandelten Erregern gibt es nach wie vor deutliche Wissenslücken zu den Übertragungswegen. Zukünftige One Health-basierte Untersuchungen werden zu deren weiterer Aufklärung und somit zur Entwicklung von Präventionsmaßnahmen beitragen. Die ganzheitliche Herangehensweise beinhaltet nicht zwangsläufig eine Fokussierung auf virale Erreger/Erkrankungen, sondern beinhaltet auch die Frage der Wechselwirkungen von viralen, bakteriellen und anderen Erregern, inkl. der Antibiotikaresistenz und der Wirtsmikrobiome.

## Abstract (en)

The COVID-19 pandemic and the increasing occurrence of monkeypox (mpox) diseases outside Africa have illustrated the vulnerability of populations to zoonotic pathogens. In addition, other viral zoonotic pathogens have gained importance in recent years.

This review article addresses six notifiable viral zoonotic pathogens as examples to highlight the need for the One Health approach in order to understand the epidemiology of the diseases and to derive recommendations for action by the public health service. The importance of environmental factors, reservoirs, and vectors is emphasized, the diseases in livestock and wildlife are analyzed, and the occurrence and frequency of diseases in the population are described. The pathogens selected here differ in their reservoirs and the role of vectors for transmission, the impact of infections on farm animals, and the disease patterns observed in humans. In addition to zoonotic pathogens that have been known in Germany for a long time or were introduced recently, pathogens whose zoonotic potential has only lately been shown are also considered.

For the pathogens discussed here, there are still large knowledge gaps regarding the transmission routes. Future One Health-based studies must contribute to the further elucidation of their transmission routes and the development of prevention measures. The holistic approach does not necessarily include a focus on viral pathogens/diseases, but also includes the question of the interaction of viral, bacterial, and other pathogens, including antibiotic resistance and host microbiomes.

## Vorbemerkung

One Health ist ein „kollektiver, vereinender Ansatz, der darauf abzielt, die Gesundheit von Menschen, Tieren und Ökosystemen nachhaltig ins Gleichgewicht zu bringen und zu optimieren. Er erkennt an, dass die Gesundheit von Menschen, Haus- und Wildtieren, Pflanzen und der weiteren Umwelt (einschließlich der Ökosysteme) eng miteinander verbunden und voneinander abhängig sind. Der Ansatz mobilisiert verschiedene Sektoren, Disziplinen und Gemeinschaften auf unterschiedlichen Ebenen der Gesellschaft“ [1]. Sektorübergreifend bedeutet in diesem Zusammenhang, dass die Zusammenarbeit und der Austausch zwischen verschiedenen Sektoren, wie z. B. Gesundheitswesen, Veterinärmedizin, Umweltschutz, Landwirtschaft und Wissenschaft, notwendig sind, um eine umfassende Sicht auf die Gesundheit zu gewährleisten und effektive Lösungen für Gesundheitsprobleme zu entwickeln. Darüber hinaus beinhaltet es eine Zusammenarbeit verschiedener Regierungsbehörden, Nichtregierungsorganisationen (NGOs), internationaler Organisationen und der Privatwirtschaft, um gemeinsame Ziele im Bereich der Gesundheit zu erreichen. Durch diese sektorübergreifende Zusammenarbeit können Synergien geschaffen werden, um Gesundheitsprobleme zu lösen und eine nachhaltige Gesundheitsentwicklung zu fördern.

**Figure 1:**
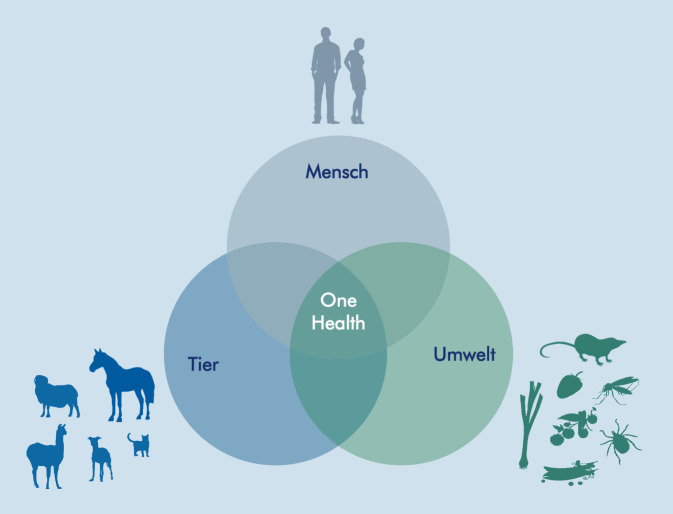

Bei der Erforschung, Prävention sowie Bekämpfung von viralen Zoonosen ist es nicht zielführend, einen isolierten Blick auf die Erkrankung beim Menschen oder bei Tieren, einschließlich der tierischen Erreger-Reservoir-Systeme zu werfen. Vielmehr sollte hierbei – wie im vorliegenden Beitrag beschrieben – der holistische „One Health-Ansatz“ zur Anwendung kommen. Der Begriff „Tiergesundheit“ umfasst Nutz- und Wildtiere. „Umweltgesundheit“ umfasst die abiotische und biotische Umwelt, inkl. der Kleinsäugerreservoirwirte, wie Nagetiere und Spitzmäuse, und Vektoren, wie Zecken und Stechmücken, die an der Erregerübertragung beteiligt sind (Abb. 1).

Charakteristisch für virale Zoonosen ist die dauerhafte Verbreitung des Erregers in bestimmten Tierspezies, den sogenannten Reservoirwirten. In Reservoirwirten führt die Infektion aber nicht immer zu einer Erkrankung. Reservoirwirte tolerieren die Infektion häufig, ohne eine klinische Symptomatik zu entwickeln, da der Beziehung Erreger-Reservoirwirt vielfach eine lange Koevolutionsgeschichte vorausgegangen ist. Springt der Erreger nun auf eine andere Spezies als den Reservoirwirt über (z. B. den Menschen), so wird das als Spillover bezeichnet, der neue Wirt auch als „Fehlwirt“ (Dead-end Host). Findet ein solches Spillover-Event statt, so kann dies ganz unterschiedliche Folgen für den Fehlwirt haben, die von Infektionsresistenz bis hin zu schweren oder letalen Krankheitsverläufen reichen können [2–5].

## Einleitung

Die COVID-19-Pandemie und das erstmals auch in Europa gehäufte Auftreten von Mpox-Erkrankungen (ursprünglich: Affenpocken) haben die Verletzlichkeit der Bevölkerung für aus dem Tierreich stammende Krankheitserreger deutlich werden lassen [6–9]. Darüber hinaus haben in den vergangenen Jahren weitere virale Zoonoseerreger große Aufmerksamkeit erfahren: So wurde im Jahr 2022 in einer kleinen Gemeinde in Bayern erstmalig eine lokale Häufung von letalen Enzephalitiden bekannt, die auf Infektionen mit dem Borna Disease Virus 1 (BoDV-1) zurückgeführt werden konnten [10]. Auch die Zahl der erfassten Hepatitis E-Erkrankungsfälle ist in den vergangenen Jahren in Deutschland fast kontinuierlich angestiegen [11]. In regelmäßigen Abständen treten zudem regionale Häufungen von Hantavirus-Erkrankungsfällen (bekannt als so genannte „Ausbruchsjahre“) auf [12]; kürzlich wurde sogar von einer Hantavirus-Erkrankung durch Heimratten berichtet [13]. Seit 2018 kommt das von Mücken übertragene West-Nil-Virus (WNV) in bestimmten Regionen Deutschlands vor (vorrangig in den östlichen Gebieten) und hat sich dort fest etabliert [14]. Außerdem nehmen Erkrankungen durch das Frühsommer-Meningoenzephalitis-Virus (FSME-Virus) in Deutschland seit Jahren kontinuierlich zu. Das Verbreitungsgebiet des Erregers dehnt sich dabei nach Norden und auch im Osten Deutschlands aus. Auch lebensmittelübertragene FSME-Erkrankungen sind kürzlich in die Schlagzeilen geraten [15]. Hinzu kommen vereinzelte Spillover-Infektionen vom Reservoir auf andere Tierarten, die jedoch ohne oder nur mit begrenzter Zirkulation in der jeweiligen Tierart verbunden sind [16], wie der aktuelle Bericht über eine letale Infektion eines Affen in einem zoologischen Garten mit dem lymphozytären Choriomeningitis-Virus (LCMV) veranschaulicht [17].

Andere für den Menschen gefährliche zoonotische Viren, wie die Lyssaviren (Tollwutvirus und Fledermaus-Tollwutviren) führen derzeit glücklicherweise nur sehr selten zu humanen Infektionen in Deutschland, beziehungsweise werden nur als aus dem Ausland importierte Fälle berichtet [18]. Eine besondere Bedeutung haben die zoonotischen Influenzaviren des Vogels (aviäre Influenzaviren) und des Schweins (Schweineinfluenzaviren), insbesondere auch als Vorläufer von pandemischen Influenzaviren. Während in Deutschland glücklicherweise noch keine humanen Fälle mit aviären Influenzaviren beobachtet wurden, kommt es vereinzelt zur Infektion des Menschen mit Schweineinfluenzaviren.

Die mit den hier aufgelisteten Zoonoseerregern verbundenen Forschungsfragen sowie die Implementierung von Bekämpfungs- und Präventionsmaßnahmen lassen sich nur in einer sektorübergreifenden, transdisziplinären Betrachtungsweise im Sinne des One Health-Konzepts erfolgreich angehen. Ziel dieses Artikels ist daher die Darstellung der Herausforderungen und Wissenslücken anhand von ausgewählten Beispielen meldepflichtiger viraler Zoonosen. Diese exemplarische Darstellung beinhaltet Hantaviren, Hepeviren, das West-Nil-Virus, das FSME-Virus, zoonotische Influenzaviren und Bornaviren. All diese Viren und die dadurch induzierten Erkrankungen reflektieren unterschiedliche Übertragungswege, verschiedene Erreger-Diversitäten und Reservoire sowie unterschiedliche Erkrankungen bei Mensch und Tier (Tab. 1).

**Table 1:** 

Erreger (Anzahl Erregerarten in DE)	Erkrankung	Symptome	Humane Meldefälle in DE (*n*)	Letalität	Erkrankung bei Tieren	Übertragung	Umweltstabilität	Referenz
Hantaviren (mindestens 9; Tab. 2)	*In Europa und Asien:* Hämorrhagisches Fieber mit renalem Syndrom (HFRS) *In Amerika:* Hantavirus-induziertes kardiopulmonales Syndrom (HCPS)	Grippeähnliche Symptome, Fieber, Blutungen, Niereninsuffizienz, Proteinurie, Anurie, Schock	Fallzahl schwankt jährlich. Im Zeitraum 2018–2022 wurden durchschnittlich 780 Fälle übermittelt (Range: 141–1719)	HFRS: 0,1–12,0 % HCPS: bis zu 35 %	Symptomlos im Reservoir	*Indirekt:* durch Aufwirbeln virushaltiger Stäube von Reservoirexkrementen *Direkt*: durch Biss	Unbekannt, unter experimentellen Bedingungen 10 bis 15 Tage bei Raumtemperatur; bei 4 °C bis zu 18 Tage	[11, 30, 118]
Hepatitis E-Virus und verwandte Viren (2; Tab. 3)	Hepatitis E	Akut: grippeähnliche Symptome, Oberbauchschmerzen, Ikterus Chronisch: Leberzirrhose	Von 2011 bis 2018 stark ansteigende Fallzahlen (von 238 auf 3400 Fälle). Seit 2018 relativ stabile Fallzahlen mit durchschnittlich 3400 Fällen pro Jahr (Range: 3079–3729)	0,5–4 %	Symptomlos	Hauptsächlich durch den Verzehr ungenügend erhitzter Fleischprodukte von Haus- und Wildschwein. Auch über kontaminiertes Trinkwasser, Blutprodukte und direkten Tierkontakt möglich	Sehr stabil bei pH 2–9 und Salzkonzentrationen bis 20 % NaCl; stabil nach Trocknung (nur 1 log Verringerung nach 8 Wochen auf Plastik bei 4 °C, stärkere Inaktivierung auf Holz)	[11, 40, 119, 120]
Bornaviren (2; Tab. 4)	Progressive Enzephalitis	Fieber, Kopfschmerzen, Sprach- und Koordinationsstörungen, Koma	Erst seit März 2020 besteht eine Meldepflicht. Zwischen 2020–2022 sind dem RKI jährlich 5–7 akute Infektionen mit Borna Disease Virus 1 (BoDV-1) übermittelt worden	> 95 %	Borna’sche Krankheit bei Haustieren, insbes. Pferden, Schafen und Neuweltkameliden	BoDV‑1 durch Spitzmäuse, Variegated Squirrel Bornavirus 1 (VSBV-1) durch gehaltene exotische Hörnchen; genauer Übertragungsweg unbekannt	Keine genauen Daten verfügbar, aber vermutlich gering	[63, 64, 121]
West-Nil-Virus (WNV; 1)	West-Nil-Fieber, West-Nil-Enzephalitis	Grippeähnliche Allgemeinsymptomatik, Kopfschmerz, Übelkeit, Erbrechen, Nackensteifigkeit, Bewusstseinsänderung, Koordinationsstörungen, Lähmungen; schwere Verlaufsformen im höheren Alter gehäuft	2018 sind autochthone Übertragungen von WNV erstmals in DE nachgewiesen worden. Im Zeitraum 2018–2022 wurden jährlich 5–21 WNV-Fälle an das RKI übermittelt, von denen ein Teil reiseassoziiert war	Bei Enzephalitis im höheren Alter bis 10 %	Meningitis, Enzephalitis bei Pferden	Stechmückenstiche; selten Kontakt mit virushaltigem Blut, Gewebe	Keine genauen Daten verfügbar, aber vermutlich gering	[18, 96, 100]
Frühsommer-Meningoenzephalitis-(FSME-)Virus (1); *5 Subtypen, davon der europäische, sibirische und fernöstliche Subtyp medizinisch am wichtigsten*	In Europa und Nordasien Meningitis, Enzephalitis, Enzephalomyelitis	Grippeähnliche Allgemeinsymptomatik, Kopfschmerz, Übelkeit, Erbrechen, Nackensteifigkeit, Bewusstseinsänderung, Koordinationsstörungen, Lähmungen	Jährlich schwankende Fallzahlen. Im Rekordjahr 2020 sind > 700 Fälle an das RKI übermittelt worden (Durchschnittl. FSME-Fallzahl 2018–2022: 540)	Europäischer Subtyp: 1–2 %; sibirischer Subtyp: ca. 5 %; fernöstlicher Subtyp: bis ca. 20 %	Meningitis, Enzephalitis bei Pferden, Affen, selten bei Hunden, vereinzelt bei Schafen	Zeckenstiche, selten Konsum roher Milch/Milchprodukte von infizierten Ziegen, Schafen, Kühen	Keine genauen Daten verfügbar; bei 4 °C in proteinhaltigen Lösungen (z. B. Milch) bis zu 2–3 Wochen	[11, 15, 78]
Lymphozytäres Choriomeningitis-Virus (1)	Verschiedene Krankheitsbilder darunter Meningitis, Enzephalitis	Grippeähnliche Allgemeinsymptomatik, Kopfschmerz, Übelkeit, Erbrechen, Nackensteifigkeit, Bewusstseinsänderung, Koordinationsstörungen, Lähmungen	Keine systematischen Angaben zu humanen Erkrankungsfällen verfügbar	Unbekannt	Häufig letale Hepatitis bei Neuweltprimaten	Übertragung über infektiöse Ausscheidungen von Hausmäusen und Hamstern	Unbekannt	[17]
Rabiesvirus (1)	Tollwut	U. a. Kopf‑, Muskelschmerzen, Schluckstörungen, vermehrte Speichelbildung, erhöhte Reiz- und Erregbarkeit, Angstzustände, Lähmungen	Seit 2005 gab es 5 Tollwuterkrankungen in DE, hiervon 2 reiseassoziierte Infektionen und 3 Infektionen nach Erhalt eines Organs einer infizierten Spenderin	~ 100 %	Tollwut bei Säugetieren	Übertragung i. d. R. durch Bissverletzungen	gering	[18]
Zoonotische Influenzaviren (unbekannt)	Influenza-like Illness; Pneumonie	Grippeähnliche Allgemeinsymptomatik vergleichbar mit saisonaler Influenza. Bei aviärer Influenza häufig Beteiligung der unteren Atemwege	Zoonotische Influenza kommt in DE extrem selten vor. 2020 wurde ein Fall einer porcinen Influenza berichtet	Bei aviärer Influenza: 20–60 % je nach Subtyp	Geflügelpest: Je nach Virusstamm häufig letal verlaufende Erkrankung bei Vögeln und Geflügel; Schweineinfluenza: Akute Atemwegserkrankung bei Schweinen mit geringer Letalität	Übertragung durch Kontakt zu infizierten Vögeln oder Schweinen	gering	[18]

*DE* Deutschland, *RKI* Robert Koch-Institut

## Hantaviren – Erkrankungshäufungen und Nagetiermassenvermehrung

Die Entdeckung der Hantaviren geht auf Untersuchungen nach dem Koreakrieg in den 1950er-Jahren zurück [19, 20]. Im Rahmen dieser Untersuchungen in Südkorea wurde das Hantaanvirus (HTNV) in seinem Tierreservoir, der Brandmaus (*Apodemus agrarius*), entdeckt. Gegenwärtig sind insgesamt 53 Hantavirusarten beschrieben worden [21], darunter auch hochvirulente Hantavirus-Spezies wie das Sin-Nombre-Virus (SNV) mit dem Reservoirwirt Hirschmaus (*Peromyscus maniculatus*) in Nordamerika. Umfangreiche Untersuchungen zum Auftreten von SNV-Infektionen zeigten die Rolle klimatischer Besonderheiten auf. So führen das regelmäßig auftretende Wetterphänomen El Niño und die damit verbundenen starken Regenfälle zu einer Zunahme der Hirschmauspopulation [22], was letztlich zu einem nachfolgenden Anstieg der Hantavirus-Infektionen führt. Eine Übersicht zu den Charakteristika von Hantaviren und der verursachten Erkrankungen findet sich in Tab. 1.

In Deutschland werden die meisten Hantavirus-Erkrankungen durch das Puumala-Orthohantavirus (PUUV) hervorgerufen; Infektionen mit diesem Virus sind schon längere Zeit bekannt. Dieses Virus wird ausschließlich von der Rötelmaus (*Myodes glareolus*, syn. *Clethrionomys glareolus*) als Erregerreservoir auf den Menschen übertragen [23]. Im Rahmen einer intensiven Zusammenarbeit mit Partnern aus forstlichen Institutionen, dem Julius Kühn-Institut und der Universität Bern wurde die Ursache für das begrenzte Vorkommen des PUUV in bestimmten Teilen Deutschlands aufgeklärt. Mutmaßlich wurde das PUUV während der nacheiszeitlichen Wiederbesiedlung Deutschlands und Westeuropas aus einem westlichen Refugium der Rötelmaus eingeschleppt [24]. Die heterogene Verteilung des PUUV in Deutschland betrifft dabei nicht nur das Fehlen des PUUV in den Bundesländern Mecklenburg-Vorpommern, Brandenburg, Berlin, Sachsen-Anhalt, Sachsen sowie im östlichen Teil von Thüringen, sondern beispielsweise auch in Teilen Bayerns und Baden-Württembergs. Ungünstige Umweltbedingungen beeinträchtigen möglicherweise den Erhalt des PUUV in lokalen Reservoirpopulationen und können auch zum Aussterben von Viruslinien führen [25]. In Endemiegebieten wird eine Evolution des PUUV in den lokalen Rötelmauspopulationen beobachtet; dadurch lassen sich bestimmte Viruslinien mit ihrer geografischen Verbreitung assoziieren [26–28]. Die Häufung von humanen Erkrankungen in bestimmten Jahren ist eng verknüpft mit einer starken Vermehrung der Rötelmaus. Diese Prozesse werden in Mittel- und Westeuropa durch die „Buchenmast“, eine starke Fruchtbildung bei der Buche (*Fagus sylvatica*), getrieben [29]. In Deutschland wurden solche „Ausbruchsjahre“ in den vergangenen Jahren im Zweijahresrhythmus beobachtet [11].

**Figure 2:**
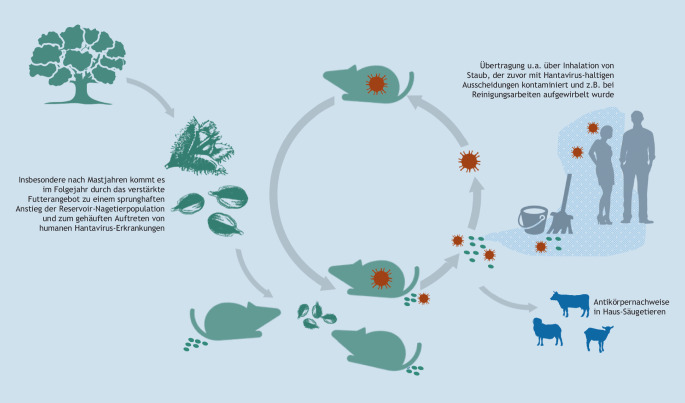

Die Übertragung des PUUV und vermutlich aller mit Säugetieren assoziierten Hantaviren erfolgt vor allem indirekt, z. B. durch das Einatmen kot- oder urinkontaminierten Staubs (Abb. 2). Unter Laborbedingungen bleibt die Tenazität des Hantavirus über mehrere Wochen erhalten [30]. Darüber hinaus geht man von einer Übertragung durch Bisse infizierter Nagetiere aus [31]. Weitere humane Infektionen sind in Deutschland auf die Hantavirusarten Dobrava-Belgrad-Virus (DOBV), Genotyp Kurkino, mit der Brandmaus (*Apodemus agrarius*) als Reservoir, das Tulavirus mit der Feldmaus (*Microtus arvalis*) als bevorzugtem Reservoir und ein Heimratten-assoziiertes Seoulvirus (SEOV) zurückgeführt worden [13, 32, 33]. Neben diesen Nagetier-assoziierten zoonotischen Hantaviren sind in den vergangenen Jahren weitere Hantaviren in anderen Nagetieren, aber auch in Spitzmäusen, Maulwürfen und Fledermäusen entdeckt worden (Tab. 2).

**Table 2:** 

Hantavirus	Reservoir	Zoonotisch	Verbreitung in Deutschland	Referenz
Puumalavirus	Rötelmaus (*Myodes glareolus*, syn. *Clethrionomys glareolus*)	Ja	Westliches, südliches und nordwestliches Deutschland	[24, 26, 27]
Dobrava-Belgrad-Virus	Brandmaus (*Apodemus agrarius*)	Ja	Östlicher Teil Deutschlands	[32, 122, 123]
Tulavirus	Feldmaus^a^ (*Microtus arvalis*)	Ja	Gesamtes Deutschland	[33, 124]
Seoulvirus	Wanderratte (*Rattus norvegicus*)	Ja	Heimratten, nicht in Wildratten	[13, 125]
Tatenalevirus, Stamm Traemmerseevirus	Erdmaus (*Microtus agrestis*)	Unbekannt	Ein Nachweis in Brandenburg	[126]
Seewisvirus	Waldspitzmaus (*Sorex araneus*)	Unbekannt	Deutschlandweit	[127, 128]
Asikkalavirus	Zwergspitzmaus (*Sorex minutus*)	Unbekannt	An einem Fangort	[129]
Brugesvirus	Europäischer Maulwurf (*Talpa europaea*)	Unbekannt	An einem Fangort	[130]
Brnovirus	Großer Abendsegler (*Nyctalus noctula*)	Unbekannt	An 3 Orten	[131]

^a^Das Tulavirus wurde auch in verwandten Wühlmausarten molekular nachgewiesen, z. B. in der Erdmaus (*Microtus agrestis*); möglicherweise stellt die Erdmaus auch einen Reservoirwirt dar

Obgleich bei serologischen Studien Hantavirus-reaktive Antikörper in verschiedenen Haus- und Nutztieren nachgewiesen worden sind, gibt es bisher keine Hinweise auf Erkrankungen bei diesen Tieren [34].

## Hepatitis E-Virus – Ein lebensmittelübertragener Zoonoseerreger

**Figure 3:**
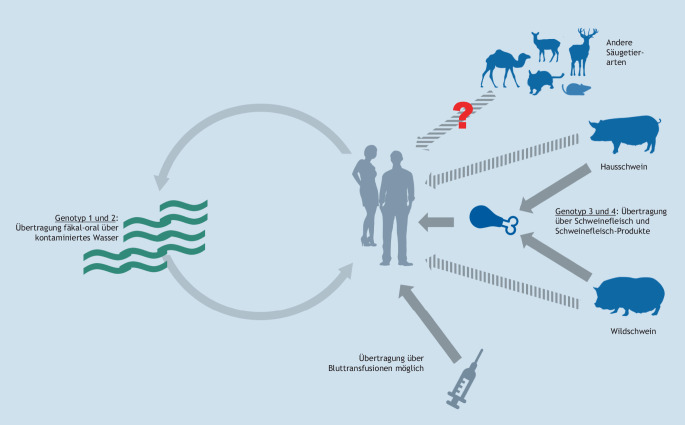

Das humanpathogene Hepatitis E-Virus (HEV, Spezies *Paslahepevirus balayani*; Familie *Hepeviridae*; [35]), wurde erstmals 1983 durch einen Selbstversuch nachgewiesen, bei dem sich der Arzt Mikhail Balayan eine filtrierte Stuhlprobe eines an einer neuartigen Hepatitis Erkrankten verabreichte [36]. Ab 1997 wurden auch in Schweinen HEV-Stämme gefunden, die denen des Menschen sehr ähnlich waren [37]. Experimentelle Infektionen von Haus- und Wildschweinen sowie molekularepidemiologische Nachweise von Virusübertragungen durch Fleisch- und Wurstwaren auf den Menschen wiesen später den zoonotischen Charakter der HEV-Infektion nach ([38]; Abb. 3). Eine zoonotische Übertragung von HEV ist vor allem auf die Genotypen HEV‑3 und HEV‑4 zurückzuführen. Daneben existieren die humanpathogenen Genotypen HEV‑1 und HEV‑2, welche nur von Mensch zu Mensch, hauptsächlich über fäkal kontaminiertes Trinkwasser, übertragen werden [38]. Weiterhin kann der zoonotische Genotyp HEV‑7 von Dromedaren auf den Menschen übertragen werden [39]. In Deutschland spielt HEV‑3 mit Abstand die größte Rolle; eine Übersicht zu weiteren hierzulande in Säugetieren vorkommenden HEV-ähnlichen Viren ist in Tab. 3 zu sehen.

**Table 3:** 

Hepevirus	Genotyp	Wirt	Zoonotisch	Geografische Verbreitung	Referenz
Humanes Hepatitis E-Virus *(Paslahepevirus balayani)*	Vor allem Genotyp 3	Wildschwein (*Sus scrofa*), Hausschwein, Kaninchen (*Oryctolagus cuniculus*) und weitere Säugetiere	Ja	Im gesamten Deutschland	[49, 119, 120]
Ratten-Hepatitis E-Virus (*Rocahepevirus ratti*)	N. d.^a^	Wanderratte (*Rattus norvegicus*)	Ja^b^	Im gesamten Deutschland	[50, 51, 132–134]
Feldmaus-Hepevirus	N. d.	Feldmaus (*Microtus arvalis*)	Unklar	An 4 Fangorten	[135]
Fledermaus-Hepeviren	N. d.	Breitflügelfledermaus (*Eptesicus serotinus*) Wasserfledermaus (*Myotis daubentonii*) Bechsteinfledermaus (*Myotis bechsteinii*)	Unklar	Einzelne positive Proben	[136]

^a^Mögliche Unterschiede zwischen den Genotypen des Ratten-Hepatitis E-Virus bezüglich des Zoonosepotenzials sind nicht bekannt

^b^Der Nachweis des Zoonosepotenzials des Ratten-Hepatitis E-Virus beruht auf dem molekularen Nachweis viraler RNA in Patienten in Hongkong, Kanada und Spanien; in Deutschland liegen bisher solche Daten nicht vor

*N*. *d*. nicht definiert

Die Infektion mit HEV kann beim Menschen eine akute Hepatitis hervorrufen. Infektionen mit HEV‑1 bei Schwangeren sowie Infektionen mit allen humanpathogenen Genotypen bei Personen mit Lebervorschädigung können zu schweren und tödlichen Erkrankungen führen. Darüber hinaus stellen chronische Infektionen bei Transplantationspatienten, die zu einer lebensbedrohlichen Leberzirrhose führen können, ein zunehmendes Problem dar [40]. Ansonsten verlaufen die meisten HEV-Infektionen aber offensichtlich mild oder ohne klinische Symptome. Die Zahl der dem Robert Koch-Institut (RKI) gemeldeten Hepatitis E-Fälle zeigte in den vergangenen 10 Jahren einen deutlichen Anstieg, der aber vermutlich auf verbesserte und intensivierte Diagnostik zurückzuführen ist (Tab. 1, [11]). Eine deutschlandweite Studie zeigte, dass bei durchschnittlich 15,3 % der Bevölkerung HEV-spezifische Antikörper nachweisbar sind [41].

Vor allem in Haus- und Wildschweinen in Deutschland, aber auch in geringerem Maße in Wildwiederkäuern, Kaninchen und anderen Tierarten, wurde HEV häufig nachgewiesen. Hierbei wurden Antikörperprävalenzen von 46,9 % in Hausschweinen [42] und 33,0 % in Wildschweinen [43] ermittelt. Die Tiere erkranken bei einer HEV-Infektion nicht, weshalb HEV-infizierte Tiere nicht bei der Schlachtung auffallen. Verschiedene Studien zeigen deshalb auch, dass in Lebensmitteln, die Schweinefleisch oder -leber enthalten, häufig HEV-RNA nachweisbar ist – so beispielsweise in 4,9 % der untersuchten Schweineleber-Proben [44], 15,0 % der Leberpaté-Proben [44] oder 20,0 % der Rohwurst-Proben [45] aus Deutschland. Stabilitätsuntersuchungen zeigen, dass eine Erhitzung der Lebensmittel das enthaltene HEV inaktivieren kann, jedoch nicht andere Arten der Haltbarmachung wie Pökeln, pH-Wert-Absenkung oder Trocknen [46–48]. Als Hauptübertragungsweg für HEV in Deutschland und Europa wird demnach der Verzehr von Fleischprodukten aus Haus- und Wildschweinen, die nicht ausreichend gegart worden sind, angesehen (Abb. 3). Andere Übertragungswege, beispielsweise über Blutprodukte, direkten Kontakt zu Tieren oder Umweltkontaminationen spielen demgegenüber wahrscheinlich eine untergeordnete Rolle.

Neuere Untersuchungen legen nahe, dass auch Kaninchen sowie Wander- und Hausratten eine Bedeutung bei der Übertragung von HEV oder HEV-ähnlichen Viren auf den Menschen haben können. In Kaninchen kommt ein besonderer Subgenotyp (HEV-3ra) vor, der auch in Deutschland schon mehrfach nachgewiesen wurde [49]. HEV-3ra wurde weltweit in Einzelfällen auch in humanen Hepatitis-Patienten nachgewiesen. In Wanderratten (*Rattus norvegicus*) aus Deutschland wurde im Jahr 2010 erstmals ein dem HEV verwandtes Virus identifiziert [50], das danach auch in vielen anderen Ländern vorgefunden wurde [51]. Wegen der fehlenden Übertragbarkeit dieses Ratten-HEV auf nichthumane Primaten durch experimentelle Inokulation wurde dieses Virus ursprünglich als nichtzoonotisch angesehen [52]. Kürzlich wurden allerdings in Hongkong, Kanada und Spanien mehrere humane Hepatitis-Fälle bekannt, die offensichtlich durch das Ratten-HEV hervorgerufen wurden [53]. Die genauen Übertragungswege der Ratten- und Kaninchenviren auf den Menschen sind bisher unbekannt und Gegenstand weiterer Untersuchungen. Ebenso muss in Zukunft geklärt werden, inwiefern HEV-ähnliche Viren, die in Feldmäusen (Ordnung Rodentia) und Fledermäusen (Ordnung Chiroptera) gefunden wurden (Tab. 3), auch ein Risiko der Übertragung auf den Menschen besitzen.

## Bornaviren – selten, aber meist tödlich

Die Borna’sche Krankheit (Borna Disease, BD) wird durch BoDV‑1 ausgelöst und wurde bereits im 19. Jahrhundert als „hitzige Kopfkrankheit“ bei Pferden beschrieben [54, 55]. Sie ist eine zumeist tödlich verlaufende neurologische Erkrankung, die mit einer nichteitrigen, durch Immunpathogenese vermittelten Enzephalitis einhergeht. Neben Pferden erkranken gehäuft auch Schafe und Neuweltkameliden (Alpakas, Lamas); grundsätzlich empfänglich für BoDV‑1 ist jedoch ein breites Spektrum von Säugetieren [54, 56, 57]. Das zoonotische Potenzial von BoDV‑1 blieb lange Zeit unklar, da Nachweise von Infektionen beim Menschen fehlten. Die seit den 1980er-Jahren diskutierten Zusammenhänge von BoDV‑1 und psychiatrischen Erkrankungen beim Menschen konnten nicht bestätigt werden bzw. stellten sich als Laborartefakte heraus [58–60].

Erst im Jahr 2018 wurde zweifelsfrei gezeigt, dass BoDV‑1 auch beim Menschen eine progressive Enzephalitis hervorrufen kann, die in mehr als 95 % der Fälle zum Tod führt [61, 62]. Symptomatisch äußert sich eine BoDV-1-Infektion beim Menschen initial meist durch Fieber und Kopfschmerzen, gefolgt von einer Reihe neurologischer Symptome (z. B. Sprach- und Koordinationsstörungen, Verwirrtheit, Wesensänderungen). Innerhalb weniger Tage bis Wochen fallen betroffene Personen in ein tiefes Koma und versterben in der Regel ([63]; Tab. 1).

Durch die Einführung einer Meldepflicht für Bornavirus-Infektionen bei Mensch und Tier im März 2020 ist zu erwarten, dass sich ein genaueres Bild zur Häufigkeit von humanen Erkrankungen abzeichnen wird. Zwischen 2020–2022 sind dem RKI jährlich 6–7 akute Fälle von BoDV-1-Enzephalitis übermittelt worden. Mit Stand Februar 2023 sind insgesamt 45 humane BoDV-1-Fälle erfasst worden, von denen ein Teil aus asserviertem Gewebematerial von Enzephalitisfällen unklarer Genese retrospektiv diagnostiziert wurde [63–65]. Auf Basis dieser Zahl ist von einer sehr niedrigen Inzidenz auszugehen. Momentan kann aber noch keine Aussage zum Manifestationsindex und zur entsprechenden Dunkelziffer getroffen werden: In Seroprävalenzstudien mit mehreren Hundert Teilnehmenden konnte lediglich eine einzelne seropositive Probe bei einer Tierärztin von der Schwäbischen Alb gefunden werden (u. a. [66, 67]).

**Figure 4:**
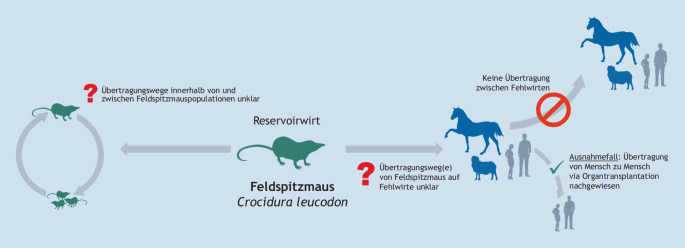

Als Reservoirwirt von BoDV‑1 wird gegenwärtig die Feldspitzmaus (*Crocidura leucodon*) angesehen, welche zu den Insektenfressern (Ordnung Eulipotyphla) gehört ([68, 69]; Tab. 4). Vermutlich tragen die Tiere das Virus lebenslang in sich, ohne daran zu erkranken, und scheiden es u. a. über Speichel, Urin, Kot und über die Haut aus [70]. Die genauen Übertragungswege innerhalb der Spitzmauspopulation sowie auf andere Wirte, wie Haussäugetiere oder den Menschen, sind bisher unbekannt (Abb. 4). In Nichtreservoirwirten verhält sich das Virus strikt neurotrop. Es ist praktisch ausschließlich im zentralen Nervensystem (ZNS) sowie vereinzelt in Nervenzellen außerhalb des ZNS zu finden und wird daher von diesen Wirten auch nicht ausgeschieden. Sie fungieren somit als Fehlwirt für das Virus. Das bisher bekannte Endemiegebiet wird durch den BoDV-1-RNA-Nachweis primär in Reservoirwirten und Haussäugetieren definiert und erstreckt sich von Bayern, über Thüringen, Sachsen, Sachsen-Anhalt bis nach Brandenburg. Auch in der Ostschweiz, sowie Teilen Liechtensteins und Österreichs kommt BoDV‑1 vor [58]. Umfangreiche Untersuchungen sind notwendig, um die exakte geografische Verbreitung des Virus festzustellen, wie eine Studie im Nordwesten Brandenburgs zeigte. Erstmalig wurde hier ein Vorkommen von BoDV‑1 in dieser Region anhand von Erkrankungsfällen bei Alpakas sowie dem Nachweis von BoDV-1-RNA in einer Feldspitzmaus belegt [56].

**Table 4:** 

Art	Wirt	Zoonotisch	Erkrankung bei Tieren	Geografische Verbreitung in Deutschland	Referenz
Borna Disease Virus 1 (BoDV-1)	Feldspitzmaus *(Crocidura leucodon)*	Ja	Ja	Bayern, Thüringen, Sachsen, Sachsen-Anhalt, Brandenburg sowie z. T. angrenzende Bundesländer	[68, 69]
Bunthörnchen-Bornavirus 1 (Variegated Squirrel Bornavirus 1, VSBV-1)	Verschiedene exotische Hörnchenarten (Virus nur in Haltungen gefunden)	Ja	Bisher unbekannt	Nur bei Hörnchen-Haltungen in Deutschland; letzter Nachweis 2019	[71, 121, 137]

Das Auftreten von 2 tödlichen Erkrankungsfällen in einer Gemeinde in Bayern 2019 und 2022 führte zu umfangreichen One Health-basierten Untersuchungen zu BoDV‑1, die sowohl die Bevölkerung als auch potenzielle Kleinsäugerwirte, Bodenproben und Zecken beinhalteten [10]. Ein Fokus zukünftiger Untersuchungen sollte auf der weiteren Charakterisierung des Erreger-Reservoir-Systems, der Stabilität des Erregers in der Umwelt sowie der Ermittlung möglicher Übertragungswege liegen. So können zukünftig präzisere Empfehlungen zur Vermeidung dieser tödlichen Infektion gegeben werden.

Neben BoDV‑1 gibt es mit dem Bunthörnchen-Bornavirus (Variegated Squirrel Bornavirus 1, VSBV-1) ein weiteres zoonotisches Bornavirus (Tab. 1 und 4). Dieses Virus wurde erstmals 2015 im Rahmen der Ermittlungen zu 3 tödlichen humanen Enzephalitisfällen beschrieben [71]. Die Entdeckung dieses Virus ist auf eine intensive Zusammenarbeit von Human- und Veterinärmedizin zurückzuführen [71], bei der eine Verbindung zwischen den humanen Fällen aufgrund der Gemeinsamkeit der Haltung von exotischen Hörnchen hergestellt werden konnte. Die darauffolgende Beprobung eines Bunthörnchens (*Sciurus variegatoides*) und der Virusnachweis mittels Hochdurchsatzsequenzierung führten zur Entdeckung des neuen Erregers. Öffentlichkeitsarbeit in Kombination mit dem Angebot einer kostenlosen Diagnostik am Friedrich-Loeffler-Institut (FLI) und entsprechenden Biosicherheitsmaßnahmen hat vermutlich zu einer Elimination des Erregers in deutschen Hörnchen-Haltungen geführt. Aktuelle Forschungsfragen beschäftigen sich mit der Aufklärung des Übertragungsweges, der Umweltstabilität sowie des geografischen Ursprungs des Erregers. Ein Überblick über humanpathogene Bornaviren findet sich in Tab. 4.

## Frühsommer-Meningoenzephalitis-Virus – weiter auf dem Vormarsch

Das FSME-Virus gehört zur Gattung *Flavivirus* und wurde 1937 im fernen Osten der ehemaligen Sowjetunion entdeckt [72]. Dort waren insbesondere bei den Grenztruppen vermehrt Fälle einer schweren Enzephalitis (russische Frühjahr-Sommer-Enzephalitis, RSSE) aufgetreten. In den darauffolgenden Jahren wurden als Überträger die dort vorkommenden Schildzecken (Taigazecke, *Ixodes persulcatus*) identifiziert. Schon im Jahr 1931 beschrieb Schneider in Österreich ein Krankheitsbild („Schneider’sche Krankheit“), von dem wir heute annehmen können, dass es sich um die europäische Form der FSME handelte [73]. In den 1940er-Jahren wurden ähnliche Krankheitsfälle auch in anderen Teilen Europas beschrieben und resultierten in der Entdeckung des FSME-Virus in der ehemaligen Tschechoslowakei im Jahr 1948. Dort wurden in den 1950er-Jahren große Epidemien beobachtet, die allerdings nicht durch Zeckenstiche, sondern durch kontaminierte Milch von großen und kleinen Wiederkäuern verursacht wurden. Heutzutage stellen Zeckenstiche, v. a. durch die Zeckenart *Ixodes ricinus* (Gemeiner Holzbock) den wichtigsten Übertragungsweg dar [74]. Es wird davon ausgegangen, dass es nur bei ca. 5–10 % der Stiche durch infizierte Zecken zu einer symptomatischen FSME kommt, wohingegen die alimentäre Übertragungsform fast immer klinisch in Erscheinung tritt [75, 76]. In den 1990er-Jahren zeigten genetische Untersuchungen, dass mindestens 3 (nach neueren Untersuchungen mindestens 5) unterschiedliche Subtypen des FSME-Virus existieren [77]. Neben dem europäischen Subtyp wurden auch ein sibirischer und ein fernöstlicher Subtyp beschrieben, die neben einer unterschiedlichen Ökologie auch unterschiedlich schwere Verlaufsformen der Infektion beim Menschen aufweisen können ([78]; Tab. 1).

**Figure 5:**
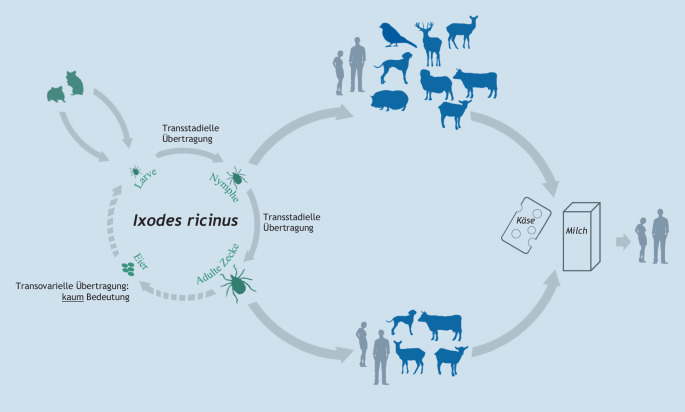

Der natürliche Übertragungszyklus des FSME-Virus vollzieht sich zwischen Zecken als natürlichen Überträgern und verschiedenen Kleinsäugerwirten. Hierzu zählen in Europa v. a. Rötelmaus, Gelbhalsmaus (*Apodemus flavicollis*) und ggf. Spitzmäuse ([79]; Abb. 5). Die natürlichen Wirte entwickeln eine ausreichend hohe Virämie, so dass blutsaugende Zecken sich wieder infizieren können. Das sog. Co-Feeding stellt eine nichtvirämische Übertragungsform dar, bei der das Virus von einer infizierten Nymphe auf eine Larve übertragen wird, die in unmittelbarer Nachbarschaft zu dieser Nymphe Blut saugt. Dabei findet eine Übertragung statt, ohne dass das Wirtstier (i. d. R. ein Nagetier) infiziert oder gar virämisch ist. Größere Waldtiere, Haustiere und der Mensch sind Fehlwirte. Sie tragen nicht zur Zirkulation des Virus in der Natur bei. Dagegen scheiden Ziegen, Schafe und Kühe das Virus über die Milch aus. Der Verzehr dieser unbehandelten viruskontaminierten Milch kann zur Infektion führen [80]. Dieser Übertragungsweg war bis in die jüngste Vergangenheit hauptsächlich in Osteuropa bekannt und war dort für bis zu 17 % der Übertragungen des FSME-Virus verantwortlich [74]. In den letzten Jahren wurde die Übertragung durch Milch vereinzelt in Deutschland, Österreich und Frankreich beobachtet, mit teilweise mehr als 40 humanen Erkrankungsfällen bei einzelnen Ausbrüchen [76, 81, 82].

Das FSME-Virus kommt in großen Teilen Europas (exklusive Spanien, Portugal) und im Norden Asiens (Russland, Mittelasien, China, Japan) vor und wurde erstmals auch in Nordafrika nachgewiesen [83]. In Europa ist die FSME eine meldepflichtige Infektionskrankheit und es werden jährlich mehrere Tausend Erkrankungsfälle registriert. Zu den Ländern mit der höchsten Inzidenz in Europa zählen Schweden, Finnland, die baltischen Staaten, Tschechien, die Slowakei, Slowenien und Österreich [84].

In Deutschland werden jährlich zwischen 350 und 700 FSME-Fälle gemeldet [11]. Davon treten ca. 85 % in den beiden Bundesländern Bayern und Baden-Württemberg auf. Auch im Bundesland Sachsen ist in den letzten Jahren ein starker Anstieg der Erkrankungsfälle zu verzeichnen. Insgesamt ist wie auch in den benachbarten Ländern Österreich, Tschechische Republik und Schweiz ein deutlich zunehmender Trend an Erkrankungsfällen zu beobachten [83]. Aktuell gibt es keine wirksame und kausale Therapie der FSME – alle therapeutischen Maßnahmen beschränken sich auf die Behandlung der Symptome. Zur Prophylaxe der FSME stehen 2 sehr wirksame und gut verträgliche Impfstoffe zur Verfügung. Die FSME-Impfung wird allen Personen empfohlen, die sich in den vom RKI ausgewiesenen Risiko-Landkreisen aufhalten und gegenüber Zecken exponiert sind [85].

Kenntnisse zur Ökologie des FSME-Virus in der Natur und zu seinem Übertragungszyklus stammen vor allem aus den 1950er- und 1960er-Jahren. Dabei zeigt sich zunehmend, dass viele der daraus resultierenden Konzepte neu überdacht werden müssen. Bisher ist der potenzielle Einfluss von Umweltfaktoren auf die Populationsdynamik der Wirte und Zecken und deren Durchseuchungsraten nur wenig verstanden. Insbesondere ist weitgehend unklar, inwieweit sich der Klimawandel auf den Übertragungszyklus des Virus, auf die Virulenz des Erregers und damit auf die Epidemiologie der Erkrankung beim Menschen auswirken wird [86]. Unverstanden sind bisher auch die Ursachen für die ungewöhnliche Epidemiologie in Deutschland (Süd vs. Nord) und den sich deutlich abzeichnenden ansteigenden Trend der Erkrankungsfälle in Mitteleuropa, darunter u. a. auch in Österreich, einem Land mit einer Impfquote von rund 85 %.

## West-Nil-Virus – weiteres Arbovirus in Deutschland seit Kurzem etabliert

Das West-Nil-Virus (WNV) gehört wie das FSME-Virus zur Gattung *Flavivirus*. Es wurde erstmals 1937 in Nordwest-Uganda, im damaligen West-Nil-Distrikt, bei einer fieberhaft erkrankten Frau isoliert [87]. In Afrika war das Virus lange ohne große Bedeutung, da in Afrika andere fieberhafte Infektionen (z. B. Malaria) die Zahl der wenigen symptomatischen WNV-Infektionen bei Weitem überstieg. In Europa wurden erst 1962 Erkrankungsfälle bei Menschen und Pferden in der Camargue (Südfrankreich) und wenig später im Süden Portugals entdeckt [88], blieben aber weiter lokal begrenzt. In den 1990er-Jahren kam es zu einigen auch größeren Ausbruchsgeschehen in Rumänien, Russland, Italien und der Tschechischen Republik. Dem Virus gelang der Sprung in die neue Welt nach New York [89]; von dort aus hat es sich innerhalb zweier Jahrzehnte nach ganz Nord‑, Mittel- und Südamerika ausgebreitet [90]. In Europa wird WNV mittlerweile in vielen südlichen Ländern nachgewiesen und ist seit 2018 auch in Deutschland endemisch. Im ostdeutschen Tiefland wird es regelmäßig nachgewiesen [91, 92].

Antigenetisch gehört das Virus, wie das ökologisch und genetisch nahverwandte Usutu-Virus, zum japanischen Enzephalitis-Komplex der Gattung *Flavivirus*. Es sind mindestens 7 genetische Linien bekannt, wobei nur die Linien 1 und 2 Erkrankungen bei Mensch und Pferd verursachen können [93].

**Figure 6:**
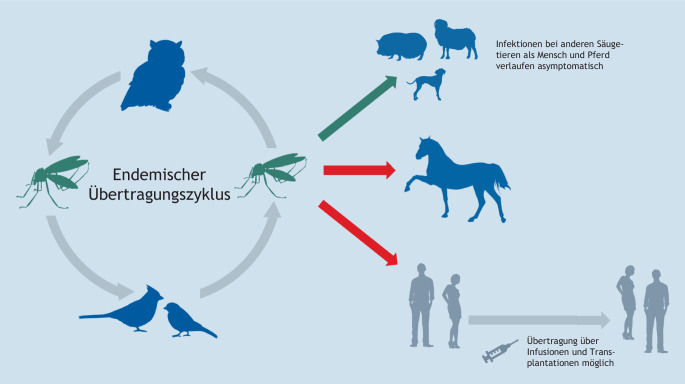

Das WNV ist ein Arbovirus, das in der Natur zwischen Stechmücken (Vektor) und Vögeln (Amplifikationswirte) zirkuliert (Abb. 6). Weibliche Stechmücken, die Wirbeltierproteine für ihre Eireifung benötigen, nehmen das Virus bei einer Blutmahlzeit von virämischen Vögeln auf und können es bei der nächsten Blutmahlzeit wieder auf andere Wirbeltierwirte übertragen. Dies sind in der Regel wiederum Vögel, so dass sich der natürliche Übertragungszyklus (enzootischer Zyklus) schließt [94]. Wenn Stechmücken sich nicht wirtsspezifisch ernähren, sondern als sog. Generalisten fungieren, können die Viren auf verschiedene Säugetiere übertragen werden. Wie in der Abb. 6 dargestellt, können dies unterschiedliche Wildtiere (z. B. Wildschweine) oder Haustiere (Hunde, Schafe) sein, die serokonvertieren, in der Regel jedoch nicht klinisch erkranken. Anders ist dies bei Pferden und Menschen. Auch hier werden keine längeren Virämien mit hohen Viruslasten erzeugt, an denen sich naive Stechmücken infizieren könnten (daher Fehlwirt), dennoch kann es zu klinisch apparenten Infektionen kommen [95].

Nach maximal zweiwöchiger Inkubationszeit kommt es bei 10–20 % der infizierten Personen zu plötzlichem Fieber mit Schüttelfrost, Kopf- und Gliederschmerzen (Tab. 1). Diese grippeähnliche Erkrankung klingt in der Regel nach ca. einer Woche ab [96]. Nur bei etwa jedem 100. Infizierten kommt es zur Infektion des ZNS mit den unterschiedlichen klinischen Ausprägungen einer Meningoenzephalitis (West Nile Neuroinvasive Disease – WNND). Diese äußert sich je nach betroffenem Hirnareal eher motorisch mit schlaffen Lähmungen und Ataxien oder mit Bewusstseinsänderungen, Gedächtnisverlust oder Verwirrtheit. Die WNND ist eine schwere Erkrankung, für die keine Kausaltherapie zur Verfügung steht und die eine Letalität von etwa 10 % aufweist [96, 97]. Impfstoffe sind bisher nur für Pferde in Deutschland zugelassen und von der Ständigen Impfkommission Veterinärmedizin (StIKo Vet) des FLI wird eine prophylaktische Impfung in den WNV-Verbreitungsgebieten empfohlen [98].

Seit dem ersten Auftreten im Jahr 2018 wurde das WNV in Deutschland regelmäßig bei Vögeln nachgewiesen [99]. Der erste humane Fall 2018 geht auf eine Infektion während der Sektion eines infizierten Vogels zurück; bei den nachfolgenden humanen Fällen wird zum einen eine Übertragung von Stechmücken, aber andererseits auch eine Übertragung im Rahmen von Blutspenden als sehr wahrscheinlich angesehen [14, 100]. Insgesamt sind mehrere Fälle von WNND in Deutschland, fast ausnahmslos bei älteren Menschen, diagnostiziert worden; letale Verläufe kamen vor [97, 100].

Derzeit ist nicht bekannt, ob es neben dem Alter und der Exposition weitere Risikofaktoren für ein West-Nil-Fieber oder eine WNND gibt. Das Wildvogel-Monitoring ist hilfreich, um frühzeitig in der Übertragungssaison das Zirkulieren von WNV zu erkennen [91, 101]. Und auch das in Deutschland durchgeführte Stechmücken-Monitoring trägt wesentlich zu unserem Verständnis bei, vor allem wo, wann, wie viele und welche Stechmückenarten Träger des Virus und damit mögliche Überträger sind [102]. Jedoch bleiben bei diesem Infektionsgeschehen noch sehr viele Fragen offen, die nur in einem One Health-Ansatz zufriedenstellend beantwortet werden können. Weitere Tierarten (Abb. 6) könnten als Sentineltiere, wie gegenwärtig noch Pferde und nach umfangreicher Durchimpfung der Pferdepopulation Wirtschaftsgeflügel in Freilandhaltung oder Zoovögel, helfen, das aktuelle Infektionsrisiko des Menschen besser zu beurteilen [103]. Die Frage, warum bei Mensch und Pferd nur ein bestimmter, zum Glück kleiner Anteil der Infizierten schwer erkrankt, impliziert bestimmte Prädispositionen. Deren Kenntnis könnte ggf. genutzt werden, diese Menschen besser zu schützen.

## Zoonotische Influenza-A-Viren als potenzielle Gefahr

Zu den zoonotischen Influenza-A-Viren (IAV) gehören bestimmte aviäre Influenzaviren (AIV), insbesondere die asiatischen hochpathogenen AIV des Subtyps H5N1 und AIV des Subtyps H7N9 in China, aber auch einige der aviären H9N2-Viren [104]. In Deutschland sind bisher keine humanen Infektionen mit AIV berichtet worden. Anders verhält es sich bei der zweiten Gruppe, den Schweineinfluenzaviren (SIV). Ein bedeutendes Beispiel der jüngeren Zeit ist das pandemische H1N1-Virus von 2009 („Schweinegrippe“). Darüber hinaus kommt es immer wieder auch zu einzelnen Spillover-Infektionen bei Menschen in Deutschland mit im Schwein endemisch vorkommenden SIV der Subtypen H1 und H3, wobei es dann oft jüngere Personen betrifft und in der Regel ein Kontakt zu Schweinen bzw. Schweinehaltungen besteht. So konnten in Deutschland in den vergangenen 3 Jahren 2 solcher Infektionen erfasst und berichtet werden [105, 106]. Die klassischen SIV mischen sich zudem seit 2009 mit dem pandemischen H1N1 und es entstehen zahlreiche neue Reassortanten, die weiter beobachtet werden müssen [107].

Das zoonotische Potenzial der aktuell in Deutschland bei Wildvögeln zirkulierenden hochpathogenen AIV vom Subtyp H5N1 (HPAIV H5N1 clade 2.3.4.4B) ist immer noch als gering einzustufen, auch wenn es in Deutschland vereinzelte Übertragungen auf Karnivoren gegeben hat und weltweit bisher 7 Fälle beim Menschen berichtet wurden [108]. Hier ist es in jedem Fall notwendig, die Situation aufmerksam weiterzuverfolgen und insbesondere weitere Übertragungsfälle auf Säugetiere genau zu untersuchen. Dies ist insbesondere vor dem Hintergrund der Berichte von ersten „Säugetier-zu-Säugetier“-Übertragungen in einer Nerzfarm in Spanien [109] und eventuell auch bei Seelöwen in Peru von Bedeutung ([110]; siehe Tab. 1).

## Fazit

Die transdisziplinäre und sektorenübergreifende Zusammenarbeit hat bei zoonotischen Erkrankungen bereits eine lange Tradition. Diese wird auch für Kleinsäuger-assoziierte Erreger durch das Netzwerk „Nagetier-übertragene Pathogene“ [111] belegt. Eine intensive Zusammenarbeit von Human- und Veterinärmedizin hat in der Vergangenheit zur erstmaligen Aufdeckung der Ursache von VSBV-1-bedingten humanen Enzephalitis-Fällen beigetragen [71]. Die One Health-Herangehensweise, bei der dann auch die Bio- und Umweltwissenschaften einbezogen werden, hat zu einem deutlich verbesserten Verständnis in der Epidemiologie viraler Zoonosen geführt. Nur durch dieses Verständnis werden die nachhaltige Bekämpfung dieser Erkrankungen und der Schutz vor Ansteckung sowie die Erarbeitung und/oder Anwendung nachhaltiger Kontrollmaßnahmen ermöglicht. Insbesondere Untersuchungen in Deutschland zu Hantaviren, Bornaviren oder FSME-Viren belegen die gemeinsame und holistische Aufklärung der Ursachen von Erkrankungshäufungen wie auch von ökologischen Prozessen. Ein besonders hervorzuhebendes Beispiel sind parallele BoDV-1-Untersuchungen von Menschen, Reservoirwirten, Vektoren und der Umwelt in Maitenbeth, einer Gemeinde in Bayern [10].

Während die Aufklärung der ätiologischen Ursachen von potenziell zoonotischen Erkrankungen voranschreitet, z. B. bei Bornaviren (siehe [71]), hat die Entdeckung „neuer“, d. h. bisher nicht bekannter Viren in Reservoirwirten zu einer erheblichen Wissenslücke bezüglich deren zoonotischen Potenzials geführt [24]. Andererseits trägt die Entdeckung bisher unbekannter Viren in den unterschiedlichen Reservoiren zu einer besseren Kenntnis der zirkulierenden Erreger bei, was eine wichtige Größe in der Prävention und Kontrolle von Infektionsgeschehen an der Mensch-Tier-Umwelt-Schnittstelle darstellt. Nichtsdestoweniger geht man gegenwärtig davon aus, dass nur ein Bruchteil der existierenden Viren bekannt ist [112]. Das betrifft beispielsweise auch Viren bei Spitzmäusen, wo erst in der jüngsten Vergangenheit eine „Virussuche“ begonnen hat [113]. Neben dem Fokus „Zoonosen“ sollten aber auch Erreger im Auge behalten werden, die bisher nur als Verursacher von Erkrankungen beim Tier erfasst worden sind und deren zoonotisches Potenzial noch unklar ist, wie z. B. beim Rustrelavirus [114–116].

Die One Health-basierten Untersuchungen berücksichtigen zunehmend auch die Frage der Folgen des Klimawandels [12]. Natürlich konzentrieren sich diese One Health-Untersuchungen nicht nur auf virale Erreger, sondern beziehen auch alle anderen Erregergruppen, wie bakterielle Erreger, inkl. der Frage der Antibiotikaresistenzen, und Endoparasiten ein. Darüber hinaus wird zunehmend auch die Rolle des Mikrobioms und die Bedeutung von Co-Infektionen, inkl. solcher mit nicht zoonotischen Viren in Kleinsäugern, berücksichtigt, um ein ganzheitliches Bild der Gefährdung von Mensch und Tier zu generieren.

Die im Rahmen des One Health-Ansatzes gewonnenen Erkenntnisse werden zu einer ganzheitlichen Herangehensweise der Bekämpfung von Infektionskrankheiten und der Aufklärung der Bevölkerung beitragen. Gleiches gilt für die Kommunikation zwischen speziellen Risiko- und Fachgruppen in Humanmedizin, Veterinärmedizin, Epidemiologie und Biologie. Für die Ärztinnen und Ärzte des öffentlichen Gesundheitsdienstes wurden hierzu bereits mehrere Workshops im Rahmen der Jahrestagung ihres Bundesverbandes durchgeführt und die Erfahrungen daraus evaluiert [117].

Da für viele zoonotische Erreger, wie z. B. Hantaviren und HEV, für Menschen in Europa noch kein Impfstoff verfügbar ist und auch in absehbarer Zeit nicht zur Verfügung stehen wird, sind andere präventive Maßnahmen zur Verhinderung notwendig. Um die Gefährdung einer Erregerübertragung durch zum Beispiel Stechmücken zu reduzieren, sollten physikalische, biologische und chemische Verfahren der Mückenbekämpfung angewandt werden. Dazu sollten u. a. stehende, künstliche Gewässer (z. B. Regentonne) regelmäßig geleert werden, um den Stechmücken kein Bruthabitat zu bieten. Zur Vermeidung von Stichen sollte lange Kleidung getragen, Mückenschutzmittel aufgetragen und die Fenster der Schlafräume mit Mückengaze verschlossen werden [100]. Zur besseren Erkennung von humanen Fällen von Zoonosen sollten Ärztinnen und Ärzte entsprechend geschult werden, so dass die gezielte Diagnostik auch angefordert wird. Nur ein gezieltes Zusammenwirken von Ärztinnen und Ärzten der Veterinär- und Humanmedizin sowie des Öffentlichen Gesundheitsdienstes (ÖGD), der Veterinärämter mit den Expertenlaboren, der mit der Mückenbekämpfung betrauten Institutionen und der Umweltwissenschaftlerinnen und -wissenschaftler wird zukünftig eine bessere Überwachung von – in vielen Fällen potenziell tödlichen – Zoonosen ermöglichen. Im Sinne des One Health-Ansatzes wurde zum Beispiel das Merkblatt „Wie vermeide ich Hantavirus-Infektionen“ unter Einbeziehung human- und veterinärmedizinischer sowie Umwelt-Expertise gemeinsam durch Robert Koch-Institut, Friedrich-Loeffler-Institut, Bernhard Nocht-Institut und Julius Kühn-Institut erarbeitet.^1^ Analog dazu wurde auch ein Merkblatt zur Vermeidung von Bornavirus-Infektionen erstellt.^2^ Das Bundesinstitut für Risikobewertung (BfR) hat zudem Fragen und Antworten zur Hepatitis E-Virus-Übertragung mit Empfehlungen zum Schutz vor Infektionen zusammengestellt.^3^

## Footnotes

1 https://www.rki.de/DE/Content/InfAZ/H/Hantavirus/Merkblatt_PDF.html [Zugriffsdatum: 30/03/2023].

2 https://www.rki.de/DE/Content/InfAZ/B/Bornavirus/Merkblatt.pdf [Zugriffsdatum: 30/03/2023].

3 https://www.bfr.bund.de/cm/343/fragen-und-antworten-zur-uebertragung-des-hepatitis-e-virus-durch-wild-und-hausschweine-und-daraus-gewonnene-lebensmittel.pdf [Zugriffsdatum: 30/03/2023].
